# Recent breakthroughs in nanostructured antiviral coating and filtration materials: a brief review

**DOI:** 10.1039/d2ra01567f

**Published:** 2022-06-01

**Authors:** Madushani H. Dahanayake, Sandya S. Athukorala, A. C. A. Jayasundera

**Affiliations:** Department of Chemistry, Faculty of Science, University of Peradeniya Sri Lanka acaj@sci.pdn.ac.lk; National Institute of Fundamental Studies Hanthana Kandy Sri Lanka; Postgraduate Institute of Science, University of Peradeniya Sri Lanka; Division of Mathematics and Science, Missouri Valley College Marshall MO 65340 USA

## Abstract

COVID-19 persists as the most challenging pandemic of the 21^st^ century with a high rate of transmission. The main pathway of SARS-CoV-2 transmission is aerosol-mediated infection transfer through virus-laden droplets that are expelled by infected people, whereas indirect transmission occurs when contact is made with a contaminated surface. This mini review delivers an overview of the current state of knowledge, research directions, and applications by examining the most recent developments in antiviral surface coatings and filters and analyzing their efficiencies. Reusable masks and other personal protective devices with antiviral properties and self-decontamination could be valuable tools in the fight against viral spread. Moreover, antiviral surface coatings that repel pathogens by preventing adhesion or neutralize pathogens with self-sanitizing ability are assumed to be the most desirable for terminating indirect transmission of viruses. Although many nanomaterials have shown high antiviral capacities, additional research is unquestionably required to develop next-generation antiviral agents with unique characteristics to face future viral outbreaks.

## Abstract 2

Types of antiviral nanofilters and coatings and their applications.

## Graphical abstract

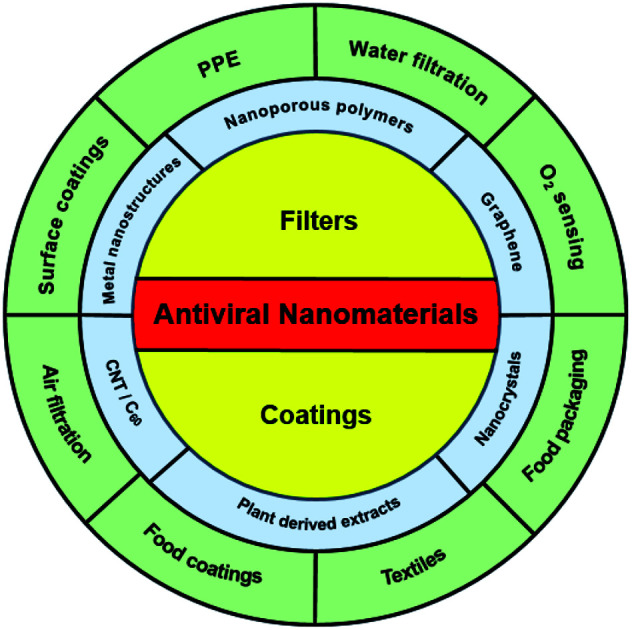

## 1. Introduction

Coronavirus disease 2019 (COVID-19) persists as the most challenging pandemic of the 21^st^ century, and, with its high rate of reproduction and transmission, it has caused 258 830 438 confirmed cases and more than 5 million deaths (as of November 24, 2021).^1,2^ Moreover, new virus strains have evolved because of mutations, and these are resistant to vaccines that target the original strain, or they have increased the virulence of the virus.^3^ Materials scientists have been working to find ways to prevent the virus from spreading in this dire situation, even in the absence of specific vaccines, therapeutics, or antimicrobial agents.^4^ The first line of defense when it comes to battling outbreaks and pandemics is to reduce viral propagation, but the COVID-19 pandemic reveals how difficult this is on a global scale.^5^

Studies show that the main pathway of severe acute respiratory syndrome coronavirus 2 (SARS-CoV-2) transmission is aerosol-mediated infection transfer through virus-laden droplets (that may be smaller or larger than 50 nm in size) that are expelled *via* coughing or sneezing by infected people.^6,7^ Indirect contact transmission occurs when a susceptible host encounters a contaminated surface in public places such as hospitals, long-term care facilities, schools, public transportation, or businesses.^8,9^ Depending on virus characteristics and environmental conditions, contagious viruses such as SARS-CoV-2, respiratory syncytial virus (RSV), rhinovirus, and influenza can survive for days to weeks, posing a great risk for transmission.^10,11^ Moreover, it has been revealed that the characteristics of a surface play a major role in the transmission of many viruses.^12^ The duration in which a virus can persist on a surface is determined by a variety of aspects, including but not limited to the type of surface, type of virus strain, temperature, room ventilation, relative humidity, and exposure to light.^13^ The main factors contributing to surface properties are adsorption,^14,15^ porosity,^16,17^ and surface hydrophobicity.^18,19^

The development of efficient antiviral materials may need to be specifically customized according to each type of virus because their individual methods of interacting with a surface are unique. All these factors in combination should be taken into account when designing an antiviral material. Having well-defined shapes and dimensions at the nanoscale, viruses could be considered as a nanomaterial themselves.^20^ For instance, under a scanning electron microscope, the SARS-CoV-2 resembles a crown with a size of 60 to 140 nm and a height of 10 nm, which is smaller than the height of SARS (15 nm) and Middle East Respiratory Syndrome (MERS) (18.5 nm).^6^ Creating smart nanostructures on the same scale and with geometry that is similar to that of viruses can be used to foster interactions that can inhibit or inactivate virus replication.^21^ Additionally, many viruses utilize glycoproteins on their surface to attach to the host cell's molecules, and antivirals based on nanomaterials that resemble these cellular attachment points can be developed.^22^

**Fig. 1:**
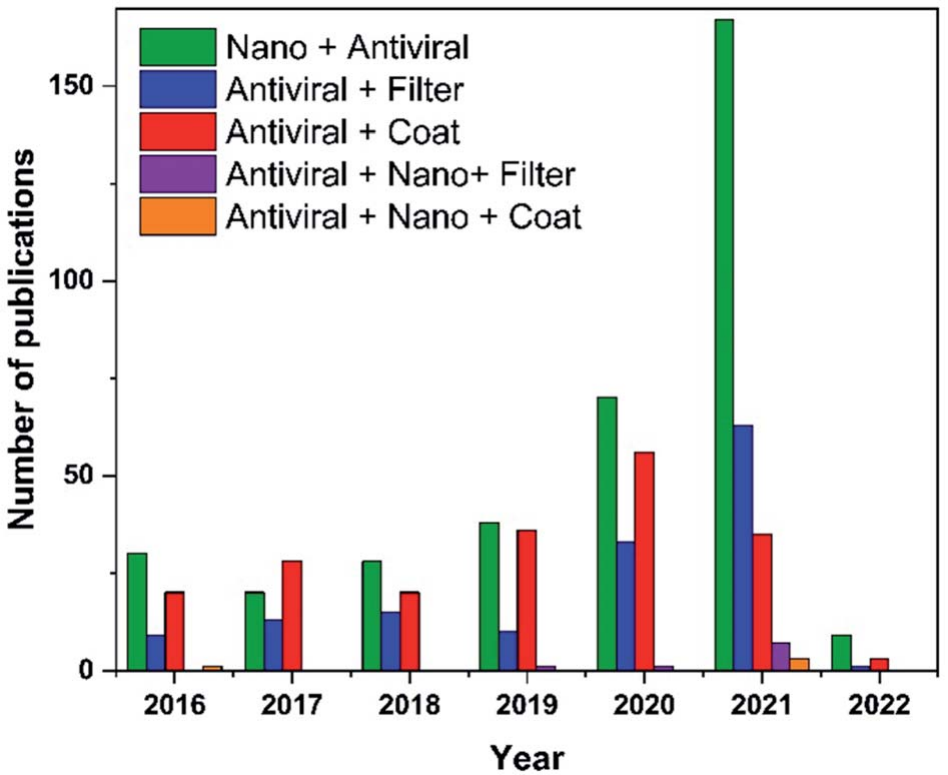
Publication trends over the last five years obtained with keywords “Nano”, “Coat”, “Filter”, “Antiviral”, and their combinations. Data analysis was completed using Scopus search system on 17 January 2022.

In this regard, recently published works show a growing interest in employing nanomaterials to fight viruses, outside and inside the host. For example, several nanoscale platforms were determined to be effective in preclinical investigations against a variety of viral infections, namely, HIV, human papilloma virus, herpes simplex, and respiratory viruses.^23^ In the past year, there has been a significant increase in the number of publications on ‘antiviral nanomaterials’ (Fig. 1). The present review provides a summary of the current state of research toward antiviral filters and antiviral surface coatings that could be applied for suppressing the evolution of viral pandemics as well as the challenges and drawbacks that require solutions.

## 2. Nanostructured antiviral filters

Filter-based protective devices are in high demand because they are efficient and easily utilized approaches for capturing airborne viruses. Coating or chemically modifying of filters are methods of enhancing their antiviral properties. It is also crucial, especially during pandemics, to produce environmentally friendly and cost-effective materials to eliminate shortages and to enable facile disposal or recycling methods.

More than 30 heavy metals are capable of interacting with microorganisms, and some possess anti-infective properties.^24^ Metal-binding/interaction mechanisms with viruses have been extensively used in the design and fabrication of antiviral filters and viral inhibitors.^25^ Copper targets the viral genome, specifically the genes that are required for viral infectivity.^8^ Many studies have incorporated Cu nanoparticles (NPs) as the antiviral agent upon surface modification of cellulose, polypropylene, polyethyleneimine, polyaniline, or nylon nonwovens and used to manufacture disposable surgical masks.

**Fig. 2:**
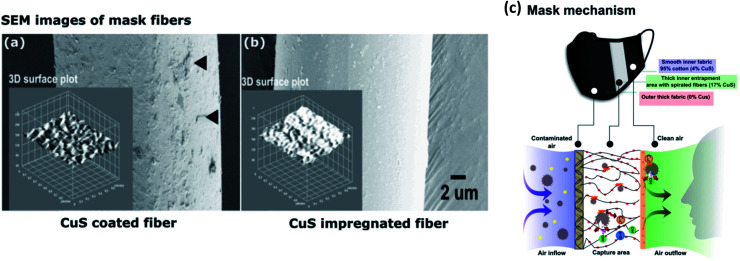
SEM images of mask fibers and virus entrapment mechanism. (a) CuS-coated fibers; black arrows indicate tightly attached CuS particles on the fiber surface. (b) CuS-impregnated mask fibers. (c) Virus inactivation mechanism of the mask (this figure has been adapted from ref. 27, with permission from Elsevier, copyright 2021).

For instance, CuI-capped *Hibiscus rosa-sinensis* L. flower extract (CuI-FE) containing a pentanoic acid and 2-(aminooxy) nanocomposite (NC) coated on cotton fabric shows high tensile strength and elongation (TS 31.58 MPa and EAB 21%, respectively) with the ability to interact with COVID-19 protease (binding energy −80.34 kcal mol^−1^).^26^ Aerosol droplets become entrapped and inactivated, and the efficiency of the face mask material was enhanced by assembling the mask barrier in three layers such that the outer two layers contained CuS NPs coated and impregnated onto 20D spandex, 70D nylon, and 75D polyester fibers that are organized in supercoiled and vertical orientations (Fig. 2). Small, aerosolized droplets are efficiently trapped, and inactivated in the middle layer by damaging the viral envelope.^27^ Furthermore, Cu deposited on a polypropylene polymer surface showed high filtration efficiency with improved adhesion when the polymer surface was treated with an oxygen ion beam, thereby forming Cu–O linkages with the Cu film that increased its capability to reduce SARS-CoV-2 nucleocapsid expression by 75%.^28^ Cu–zeolitic imidazolate framework-8 nanowires (Cu@ZIF-8 NWs) were developed by Kumar *et al.* with the aid of pluronic block copolymer, which acts as a stabilizing agent as well as a surface passivating agent with outstanding biocompatibility and reduced toxicity.^29^ Microbes are efficiently inactivated by simultaneous and sustained release of Cu and Zn ions. Additionally, the NC exhibited a lower cytotoxicity and decreased the production of pro-inflammatory cytokines and reactive oxygen species (ROS) compared to plain CuNWs, along with high thermal and chemical stability, and biocidal and self-sanitizing properties. Recent studies indicate promising results for virus removal and retention with CuNP-modified surfaces by means of electrostatic adsorption forces, with great potential for application in water purification.^30–32^ It is worth noting here that the dissolution of heavy metals in drinking water during filtration must remain within the safe limits recommended by the World Health Organization (WHO).^33^

AgNPs are in high demand due to their rapid and efficient antiviral action against a broad spectrum of viruses, including respiratory syncytial virus, norovirus, influenza virus, herpesvirus, hepatitis B virus, and human immunodeficiency virus.^34^ Moreover, AgNPs have also been used as a virucidal agent against the coronaviruses SARS-CoV-1, SARS-CoV-2,^35^ and human coronavirus HCoV-OC43.^36^ Thus, AgNPs are widely applied in personal protective equipment (PPE) development by incorporation with non-woven fibers.

**Fig. 3:**
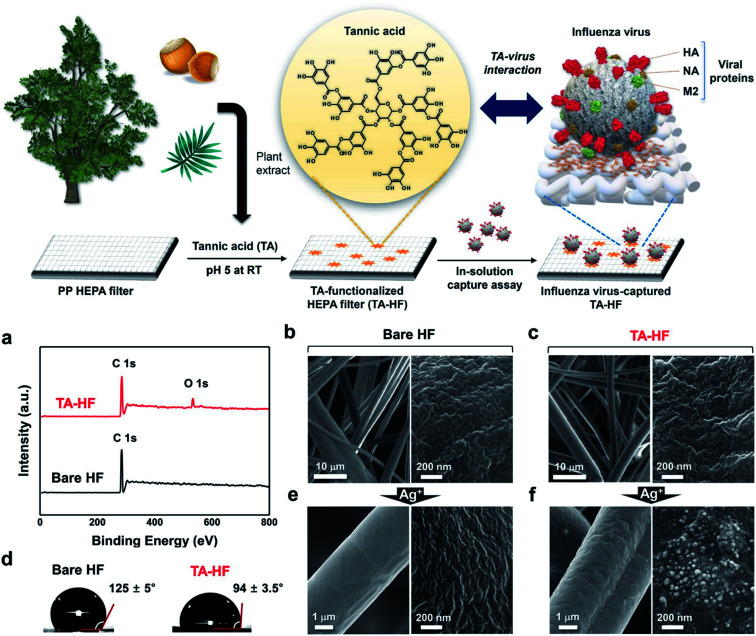
Surface functionalization of TA-HEPA filters (HF). (a) XPS spectra of TA-HF (top) and bare HF (bottom). (b) SEM images of bare HF. (c) SEM images of TA-HF. (d) Water contact angle images of the bare HF (left) and TA-HF (right). (e) SEM images of bare HF after AgNO_3_ treatment. (f) SEM images of TA-HF after AgNO_3_ treatment (this figure has been adapted from ref. 38, with permission from Springer Nature, copyright 2021).

Viral particles with smaller diameters, such as the influenza virus (approximately 100 nm), can penetrate through high-efficiency particulate air (HEPA) filters because these filters only have the ability to block the passage of particles with a diameter greater than 0.3 μm.^37^ To increase the filtration efficiency, tannic acid (TA), a plant-derived polyphenol with antiviral activity, was used for functionalization of HEPA filters as a cost-efficient adhesive with the capability of trapping viruses *via* affinity binding (Fig. 3).^38^ Furthermore, AgNP-coated HEPA filters display a decreased filtration quality factor and a decreased antiviral quality factor (0.05–0.08 Pa^−1^) with increased dust loading according to a previously established mathematical model,^39^ as dust particles tend to prevent direct contact of AgNPs with viral particles, giving rise to a decrease in the antiviral activity.^40^

*Garcinia mangostana* L. or mangosteen extract, which is a natural antimicrobial agent, and AgNPs were incorporated in a hydrophilic polyacrylonitrile (PAN)/hydrophobic polyvinylidene fluoride (PVDF) matrix to enhance its antimicrobial activity against enveloped viruses and bacteria, including tuberculosis (TB).^41^ These nanofibrous membranes possess unique characteristics, including physical and mechanical stability (TS 3.76 ± 1.08 MPa, EAB 8.67 ± 1.99%, YM 150.02 ± 32.87 MPa), wide range of antimicrobial activity, spinnability, and processability upon upscale fabrications, and the potential to be utilized in multifaceted applications, for instance, as filters for air conditioning or outdoor spaces due to their robustness in extreme weather conditions.

Palika *et al.* developed an antiviral membrane trap composed of Fe salts and amyloid nanofibrils (AFs) obtained from β-lactoglobulin (BLG) milk protein, which showed an efficiency of more than six orders of reduction of infectivity against both enveloped and non-enveloped viruses.^3^ The membrane possesses the ability of virus retention, as well as inactivation by strong interactions between positively charged iron hydroxides and negatively charged viruses. An enhanced sustainability footprint (96%) notably highlights the superiority of the membrane over conventional membranes in terms of cost, efficiency, and sustainability.

**Fig. 4:**
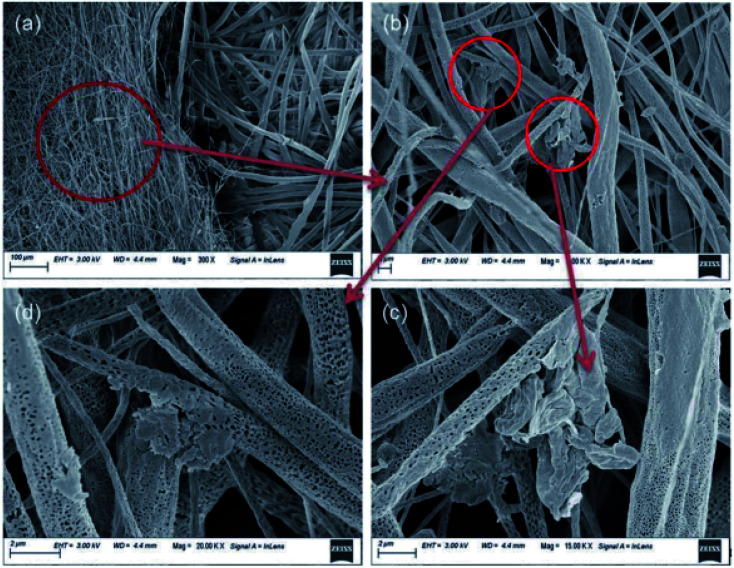
(a) SEM image of PZ15 fabric and commercial PP substrate after dipping in ethanol for 24 h. (b) SEM image of the distribution of ZL and SNC on fabric. (c) and (d) Magnified SEM images of ZL and SNC on fabric (this figure has been adapted from ref. 42, with permission from the Royal Society of Chemistry, copyright 2021).

In a novel study, it was noted that Zn oligo-lactate (ZL)-functionalized poly(lactic acid)/silk nanocrystals (SNC) on PZ15 fabric were beneficial because the material can be used as a face covering throughout the pandemic if standard PPE should become unavailable (Fig. 4).^42^ Natural muga-silk was employed to synthesize silk nanocrystals, which act as a barrier and prevent penetration and adsorption of water or moisture due to their hydrophobicity. This antiviral nanofiber matrix possesses excellent features including reusability and biodegradability, as well as sustainability.

Another recent study presented a nanoceutical cotton fabric filter coated with ZnO nanoflowers (NFs) for potential use as a one-way valve membrane with improved breathability for controlling the spread of COVID-19 infection.^43^ An antimicrobial analysis performed with *Pseudomonas aeruginosa* (model SARS-CoV-2 mimic) revealed enhanced antimicrobial efficacy of the product. An in-depth computational study has shown that ZnO NFs with two-dimensional petals display the ability to confine SARS-CoV-2 spike proteins. They are also able to attach to angiotensin-converting enzyme-2 (ACE-2) receptors in human lung epithelial cells, resulting in denaturation of the spike proteins, which confirms the trapping and inactivation of viruses.

Nano-TiO_2_ is known to be one of the most prevalent photocatalysts due to its exceptional characteristics such as non-toxicity, chemical stability, photo-oxidation of organic compounds, long-lasting stability against photo and chemical corrosion, and resilient oxidizing power under ultraviolet (UV) light.^44^ TiO_2_ has recently been identified as one of the compounds with a capacity to deactivate both Gram-positive and Gram-negative bacteria,^45^ and several viral species and parasites.^46^ A research group in Thailand has employed hydroxyapatite (HA)–TiO_2_ nanocomposite as a filter with superior antiviral activity under UV exposure.^47^ According to the results of their experiment, they have proposed a reaction mechanism such that HA induces virus adsorption on the surface, and the viruses will later be decomposed by TiO_2_ upon UV irradiation. Endre's team was able to develop TiO_2_ nanowires that generate high amounts of ROS under UV light, resulting in numerous potential applications.^48^

Carbon nanotube (CNT) filters function as a strong barrier on account of their high durability, excellent hydrophobicity, and high thermal conductivity, which prevents the proliferation of viruses, including SARS-CoV-2. Even though a CNT network consists of pore sizes smaller than viruses, it retains excellent breathability and viability. The outstanding thermal conductivity of CNTs permits hyperthermic antiviral effects, as witnessed by the rapid temperature increase to 65 °C within 5 min, which offers resilient protection against viruses. The facile processability, light weight, and low cost indicate their feasibility and reusability, emphasizing the battle against the COVID-19 pandemic.^49^ CNT air filters, mechanically supported by a porous polyester substrate, exhibit HEPA-like efficiency and reduced pressure drop. The filtration system depends on an electrically conductive CNT mat with the capability of simultaneous self-sanitation through resistive heating. This active CNT hybrid membrane reveals superior filtration efficiency (HEPA H13 level) following a Darcy's law-related trend in air permeability.^50^

Carbon (C)-dot embedded nanoporous poly(vinylidene fluoride) (PVDF) membranes, developed by Singh and the team, exhibited significant features such as hydrophobicity, air permeability, breathability, excellent nano-filtration, and, most importantly, solar-induced self-sterilization *via* sunlight absorption and concomitant heat dissipation.^51^ C-dot-PVDF membranes are an inexpensive, reusable, biodegradable, and self-sterilizing platform that can be applied in viral-blocking respirators and other PPE.

There has also been interest in graphene (G)-based nanomaterials for PPE development because of their distinctive properties such as antimicrobial activity, biocompatibility, biodegradability, and also their flexibility when used in designing and manufacturing.^52,53^ However, when G or graphene oxide (GO) are embedded into polymers, they exhibit reduced antiviral activity as compared to the pure nanomaterials.^52^ This might be due to the fact that the nanoparticles are entrapped in the fibers, thus, viral deactivation by direct physical interaction cannot occur, resulting in a diminished efficiency in antiviral activity.

Furthermore, many studies have incorporated various non-metallic functional groups coated or embedded into polymers or non-woven fibers, which can be used as virucidal agents. Their filtering capability is mainly applied in PPE development,^54–58^ oxygen sensing,^59^ air purification,^60,61^ and water purification.^62–64^ These nanocomposites show exceptional properties, such as enhanced hydrophobicity, biodegradability, breathability, non-toxicity, and bioavailability, as well as antibacterial activity (Table 1).

**Table 1:** Nanostructured antiviral filtration materials

Antiviral material	Nanostructured morphology	Antiviral activity tested with	Antiviral activity	Filtration efficiency	Applications	References
Cotton/CuI/*Hibiscus rosa-sinensis* L. flower extract (CuI-FE)	Cotton fibers: diameter 5–10 μm	SARS-CoV-2	—	—	PPE	26
	CuI-FE: prismatic, mean size 552.45 nm, crystallite size 89.01 nm					
Nylon/CuS	—	SARS-CoV-2	Antiviral capacity 0.1 MOI (2 h)	—	PPE	27
PP/Cu	PP fibers: fiber diameter 22.5 ± 1.5 μm	SARS-CoV-2	—	Filtration efficiency 91.6–95.1% (1 h)	PPE	28
	Cu_2_O/CuO (3 : 1): film thickness 20 nm					
PP/zeolitic imidazolate framework-8 (ZIF-8)/Cu	Cu NWs: diameter 20–35 nm, length 5–10 μm	SARS-CoV-2	—	Filtration efficiency 95% (0.3 μm particles)	PPE	29
	Cu@ZIF-8 core–shell NWs: diameter 60–100 nm					
Nanofibrillated cellulose (NFC)/Cu	NFC: diameter 20–60 nm	MS2 bacteriophage	Antiviral capacity >5 LRV (99.9%, 20 min)		Water filtration	32
	Cu NPs: spherical; diameter 15 nm					
Polyethyleneimine (PEI)/terephthalaldehyde (TA)/Ag/Cu	PEI/TA: 5-layer thickness approximately 2.25 nm	MS2 bacteriophage	Antiviral capacity 4.5–5 LRV (30 min)	Rate of water treatment 2500 L m^−2^ h^−1^	Water filtration	30
Al_2_O_3_/Cu	Al_2_O_3_: pore sizes 1–2 and 8–12 μm	MS2 bacteriophage	Antiviral capacity 0.2–3.2 LRV (1 h)		Water filtration	31
Poly(ethylene oxide) (PEO)/Ag	PEO fibers: diameter 0.3–5 μm	HCoV-OC43	—	Filtration efficiency 95%	PPE	36
	Ag NPs: diameter 2 nm	H1N1				
PP HEPA filter/tannic acid (TA)/Ag	PP HEPA filter: fiber diameter approximately 3 μm	H1N1 (PR8)	Virus capture efficiencies: PR8 – 83% (345 PFU mm^−2^); X31 – 93% (2723 PFU mm^−2^) (10 min)		PPE	65
	Ag NPs: grain size 20–40 nm	H3N2 (X31)				
HEPA filter/AgNPs	AgNPs: diameter 11 ± 1.5 nm	MS2 bacteriophage	Antiviral efficiency 80% (500 PFU m^−3^, 15 min)	Filtration efficiency with dust loading 80–95%	PPE	40
PAN/PVDF/Ag/mangosteen (GM)	AgNPs: spherical, diameter 6.7 ± 1.7 nm	H1N1	Antiviral efficiency 99.94% (6.10 gsm filter, 1 h)		PPE	41
	PAN/PVDF/Ag/GM: fiber diameter 171 ± 34 nm, pore size 121–313 nm					
Amyloid nanofibrils (AFs)/Fe	—	Φ6	—	Filter capacity approximately 7 × 10^3^ PFU mg^−1^	Filtration membrane	3
		H1N1				
		SARS-CoV-2				
Poly(lactic acid) (PLA)/zinc (oligolactate) (ZL)/silk nanocrystals (SNCs)	PLA/ZL/SNC: fiber diameter 1.4 ± 2 μm, porosity 46–76%	NDV	Antiviral capacity 97% (10 min)		PPE	42
Cellulose/ZnO	Cotton fibers: pore size approximately 10 μm, 20 layers	*Pseudomonas aeruginosa* (model SARS-CoV-2 mimic)	—	—	PPE	43
	ZnO NFs: petal size approximately 600 × 300 nm, interplanar lattice spacing 0.25–0.26 nm					
Hydroxyapatite (HA)/TiO_2_	HA: diameter approximately 100 nm	H1N1	Antiviral capacity 2–3 LRV upon UV exposure (1 h)	—	PPE	47
	TiO_2_: anatase, diameter 80–100 nm					
TiO_2_ nanowires	TiO_2_ NWs: diameter approximately 10 nm, interplanar distance 0.354 nm	—	—	—	PPE, air filtration, air conditioning	48
SWCNTs	SWCNTs: pore size <100 nm	HCoV-OC43 (resembles SARS-CoV-2)	—	—	PPE	49
Polyester/CNTs	Polyester: pore size approximately 100 μm, thickness 0.4 mm	MHV-A59	Antiviral capacity 99.9% by thermal induction (80 °C, 30 s)	Filtration efficiency 99.99%	Air filtration	50
	CNTs: pore size 10–100 nm	AAV9				
C-dots/PVDF	PVDF/C nanoporous film: pore size approximately 49 nm, porosity 72 ± 2.4%	—	—	Air flow rate 4.5–14.5 cubic feet per min	PPE	51
PMMA/G/GO	PMMA: fiber diameter 0.75–2.71 μm	T4 bacteriophage	Antiviral efficiency 33.6–38.7% (24 h)	—	PPE	52
	G nanoplatelets: size 110 × 170 × 2 nm					
	GO nanosheets: size 1–4 μm, thickness 0.85 ± 0.12 nm					
PP/G	PP fibers: fiber diameter 10–20 μm, pore size 20 μm	SARS-CoV-2	Antiviral efficiency 100%	—	PPE	53
	G sheets: crystallite size 16.71 nm					
Non-woven fabric (NWF)/hand soap (HS)	—	Φ6	Antiviral capacity 98–100% (1 min)	—	PPE	58
		SARS-CoV-2				
PP/lignin	—	H1N1 (PR8)	Antiviral capacity 3–6 LRV (30 min)	—	PPE	54
		HCoV-229E				
		HCoV-OC43				
Polycaprolactone (PCL)/Na-polyphosphate (Na-polyP)/Ca-polyP-NP	PCL: fiber diameter 0.5–1 μm, mat thickness 280–330 μm	V-LIP (liposomes supplemented with viroporin from SARS-CoV-2)	—	—	PPE	57
	Na-polyP/Ca-polyP-NP: diameter 60–90 nm					
PP/nano-dry-salt (NDS)	PP: fiber diameter 130–190 nm	HCoV-OC43	Antiviral capacity approximately 1.7 LRV (>98%, 30 min)	—	PPE	56
	NDS: size 115.8 nm	HCoV-229E				
Poly (lactic acid) (PLA)/*Azadirachta indica* (AI)/*Eucalyptus citriodora* (EC)	PLA: fiber diameter 8.0 ± 0.2 μm, thickness 0.41 mm, pore size 20.1429 μm	—	—	Filtration efficiency 99.99%	PPE	55
Polystyrene (PS)/5,10,15,20-tetraphenylporphyrin (TPP)	PS nanofibers: diameter 100–400 nm (avg. 253 nm)	pVL-VP1	Antiviral capacity 5 × 10^7^ for MPyV and 2 × 10^5^ for pVL-VP1 (30 min)	O_2_ permeability 1.9 × 10^−13^ cm^3^ cm cm^−2^ s^−1^ Pa^−1^, O_2_ diffusion 2.8 × 10^−7^ cm^2^ s^−1^	O_2_ sensing	59
	PS NPs: diameter 30 ± 10 nm	MPyV				
Hydantoin-polyurethane (HAPU)/sulfobetaine-polyurethane (SBPU)	HAPU/SBPU fibers: fiber diameter 0.6–0.9 μm, pore size 0.8–2.8 μm	SARS-CoV-2	Antiviral capacity: 3.13–5.17 LRV (10 min)	—	Air filtration, surface coating in healthcare, PPE	61
		TGEV				
		FCV				
Nonwoven fabric/1-chloro-2,2,5,5-tetramethyl-4-imidazolidinone	—	AI H1N1	Antiviral capacity: 3–4 LRV (1 h)	—	Air filtration	60
Poly(vinyl alcohol-*co*-ethylene) (EVOH)/poly[5,5-dimethyl-3-(3′-triethoxysilylpropyl)-hydratoin] (PSPH)/Cl	EVOH grafted-PSPH nanofibers (EPNMs): fiber diameter 715–1128 nm, pore size 1.63–4.86 μm	*Escherichia coli* phages	Antiviral capacity 5 LRV (2 min)	Water flux 2000 L m^−2^ h^−1^	Water filtration	63
Nanofibrillated cellulose (NFC)/glycidyltrimethylammonium chloride (GTAC)	NFC/GTAC aerogels: pore size 0.25–10 μm	MS2 bacteriophage	Antiviral capacity 1.2–3 LRV (93.6–99.9%, pH 7.0), 0.1 LRV (17.7%, pH 3.0), desorption of viruses at pH 3.0	—	Water purification	62
		Qbeta				

## 3. Nanostructured antiviral coatings

Less priority is given for the development of antiviral surfaces to prevent viral transmission due to the instant inactivation of several viruses on surfaces, incapacity of some viral species to proliferate outside the body, and lack of host cells. However, some viruses are viable on surfaces for a few hours or up to several days, and this poses a significant risk of disease transmission *via* the surface route. Thus, there is an urgent need for developing low-cost sustainable technology solutions that prevent virus survival on surfaces and control disease spread.^11,66^

More recently, numerous approaches have been employed to fabricate antiviral nano coatings for several applications, from mobile phone screens to air filters.^8^ For example, by incorporating perhydrolase (AcT) into a polydopamine (PDA) matrix, Wang *et al.* created a biocatalytic composite that could be applied to a variety of surfaces. The subsequent AcT–PDA coatings drastically reduced the infectivity of a SARS-CoV-2 pseudovirus within minutes.^67^ A poly(dimethyl amino methyl) styrene-*co*-1*H*,1*H*,2*H*,2*H*-perfluorodecyl acrylate (PDP) coating on polyester fabric exhibited excellent antiviral activity against lentivirus-EGFP and satisfactory biocompatibility with NIH 3T3 fibroblast cells from mice.^68^ The positive zeta potential value of PDP-coated textiles (+23.2 ± 0.2 mV) can electrostatically interact with negatively charged bacteria and viruses, and subsequently rupture the microbial structure, resulting in microbial inactivation. Moreover, due to its highly hydrophobic and oleophobic nature, a PDP coating repels various solutions from adhering to it and prevents the attachment of contaminants.

**Fig. 5:**
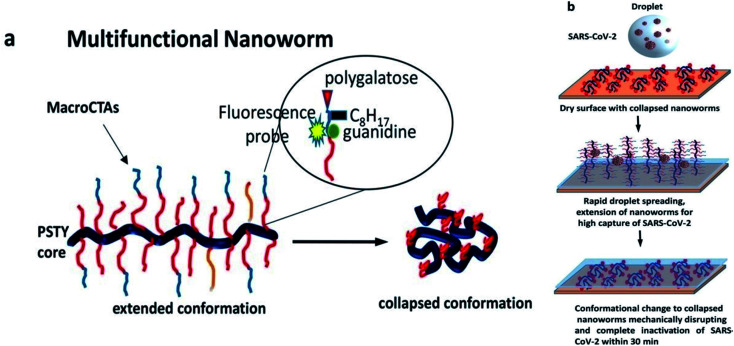
Water-based and responsive nanocoating for disruption of viruses including SARS-CoV-2. (a) Multifunctional nanoworm in extended and collapsed conformations. (b) Nanomechanical rupturing and inactivation of viruses; rapid droplet spreading resulting in extended and collapsed conformations of nanoworms due to pH change (this figure has been adapted from ref. 69, with permission from the American Chemical Society, copyright 2021).

Another prominent water-borne spray-on coating made from polystyrene and functionalized macroCTA can completely inactivate influenza A, SARS-CoV-2 (VIC01), and its alpha variant (B.1.1.7) by degrading viral RNA within 30 minutes.^69^ The large nanoscale conformational changes in this coating take place from collapsed (<100 nm) to elongated (approximately 1000 nm) upon settling of viral droplets on the surface, facilitating efficient binding and rupturing of the viral membrane (Fig. 5). Furthermore, a covalently attached fluorescent probe bound to nanoworms lower than 1500 mg m^−2^ offers a means to consider reapplication of the coating for attaining constant antiviral efficacy of the surface.

**Fig. 6:**
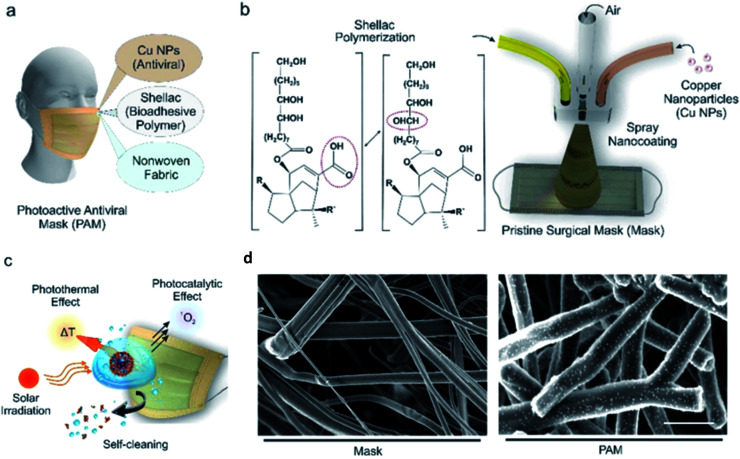
Surface modification of nanocomposite on a surgical mask. (a) Diagram showing the components of the nanocomposite coating on the surgical mask. (b) Schematic diagram of the setup for spray-coating of nanocomposite; the spray device mixes CuNPs and shellac at the junction, where pressurized N_2_ is passed through. (c) Illustration of aerosolized virus inactivation *via* photocatalytic, photothermal, and hydrophobic self-sanitizing processes. (d) SEM images (scale bar, 10 μm) of a commercial mask (left) and shellac-CuNP-coated nonwoven fibers (right) (this figure has been adapted from ref. 70, with permission from the American Chemical Society, copyright 2021).

Kumar *et al.* applied a spray-coated dual-channel hybrid nanocoating of shellac/Cu NPs to a nonwoven surgical mask, which is shown in Fig. 6.^70^ The temperature of the coated mask rapidly increased to >70 °C when exposed to sunlight, resulting in a high level of free radicals that disrupted the plasma membrane of nanosized virus-like particles. Both G and GO show excellent antiviral activities against SARS-CoV-2.^71^ After 30 minutes of contact, Das Jana *et al.* found that copper oxide (Cu_2_O) NPs in a composite with G sheets inhibited the influenza A virus.^72^ Cu_2_O/G disrupts the structural integrity of the viral envelope and the hemoagglutinin protein to impact the ability of the virus particles to enter the host cells and consequently prevents viral replication and infection.

Li *et al.* formulated a coating with long-term release-killing, contact-killing, and anti-adhesion properties, from chlorine dioxide (ClO_2_)-encapsulated micelles tethered with Cu NPs that were covalently clustered on micelle surfaces, enhancing the micelle stability and contact-killing ability.^73^ Huang's team developed a chemical modulation layer loaded with mineral acid or Cu salt-doped polyaniline to dissolve the antiviral agents in outgoing droplets that eventually became significantly concentrated, yielding dried or semi-dried respiratory nuclei, and causing deactivation of pathogens.^74^ The degree of chemical modification of droplets is approximately 0.075 at pH of 2–3, with a modification efficiency of 19–49%. Additionally, the coating tends to change its color upon depletion of acid or metal ions, which can be easily re-doped. Although these innovations can be applied in healthcare, much work remains before they can be used as a tool for infection control.

Superior antiviral performance of Ag, such as its ability to deactivate viruses through binding with the viral envelope and surface proteins, makes it an ideal candidate to generate nanocoatings that can be used for surface decontamination.^8,75,76^ For instance, Ag nanowires (NWs) that were electrospray-coated with polyacrylonitrile (PAN) fibres on their surfaces showed a significantly enhanced antiviral efficiency to 72.5 ± 1.9% in 30 minutes for bacteriophage MS2.^77^ Chen *et al.* investigated the antiviral activity of GO/Ag nanocomposites, and they proposed coating of facemasks with GO/Ag NPs to reduce the risk of infectious disease transmission.^75^ Most recently, Cox and colleagues developed a simple and inexpensive method to fabricate tea/cinnamaldehyde/Cu and tea/cinnamaldehyde/Ag hybrid nanocoatings (approximately 150 nm thickness) that spontaneously adhere to substrate material surfaces.^11^ Interestingly, a nonwoven polypropylene coating containing tea/cinnamaldehyde/copper and tea/cinnamaldehyde/silver resulted in 98.6 and 99.8% murine coronavirus deactivation, respectively.

A group of researchers synthesized a coating consisting of 26 ± 2 nm Ag NPs, 212 ± 16 nm Cu NPs and Cu particles (1.3 ± 0.2 μm) containing 51 ± 2 nm Ag NPs.^78^ Rapid inactivation of SARS-CoV-2 on the Cu/Ag nanocoating was observed only after 1 and 5 min with high volumes of Cu (65 and 78 wt%) and lower volumes of Ag (7 and 9 wt%). There have been some previous studies on air filters coated with Ag NPs and SiO_2_/Ag composites that demonstrated effective antiviral behavior without altering filtration performance.^40,79^ SiO_2_/Ag composites also function as anti-viral coatings to be used as a next-generation technology to combat SARS-CoV-2.^80^ Recently, researchers further improved Ag nanocluster (NC)-embedded silica to sputter-coat fiber-based air filters with strong virucidal activity against RSV and influenza virus type A (FluVA).^81^ Prior to that, Balagna and colleagues demonstrated the virucidal effect of Ag NCs/SiO_2_ directly sputter-coated (<200 nm with Ag 1.53 at%) on an FFP3 mask, with complete inhibition of SARS-CoV-2.^82^

In another study, Wang *et al.* coated conductive Ag/Co_3_O_4_ onto a glass fiber cloth (GFC) through *in situ* combustion to manufacture an air cleaning device that could completely inhibit the pseudovirus of SARS-CoV-2 within a few minutes when a 0.05 A current was passed through at a decreased surface temperature (<50 °C).^83^ Zhong and colleagues developed AgNP/graphene laser-printed N95 respirators, which exhibited exceptional superhydrophobic and photothermal properties when combined with Ag^+^ ion release upon microbial accumulation. Plasmonic heating increases the surface temperature above 80 °C within 1 min of solar irradiation, and superhydrophobicity results in self-sterilization of the material. These features in collaboration provide enhanced fortification to fight the COVID-19 pandemic.^84^

Moor and coworkers integrated fullerenes (C_70_) and Ag onto polystyrene-*block*-poly-4-vinylpyridine (PS-P4VP) copolymers in order to obtain dual functionality.^85^ C_70_ and AgNPs synergistically target bacteriophage that increase photo-generated ROS under visible light illumination. ZnO nanorods and AgNP-modified poly(methyl methacrylate) (PMMA) were utilized as an antiviral coating by Karagoz's team.^86^ The nanocomposite showed antiviral activity against both BCoV and BPIV3 viruses, along with its self-cleaning ability, reusability, and SERS-based sensing ability. Coating of PHBV18/AgNPs over PHBV3 films decreased murine norovirus (MNV) titers by 0.86 log, while no infectious feline calicivirus (FCV) were recovered after 24 hours.^87^

Hasan and colleagues fabricated randomly oriented nanostructured topography on aluminum alloy 6063 surfaces and observed a significant reduction (3–4 log_10_ after 24 h) of amount of viable RSV recovered in comparison to the control surfaces.^66^ Furthermore, the nanostructured surfaces exhibited sufficient modulus and hardness to withstand 1000 cycles at 2000 μN load for over 30 min. It was observed that hydrophobic sintered ceramic with La_2_Mo_2_O_9_ (LMO) powder decreased the rates of survival of bacteriophage Qβ and bacteriophage Φ6 by more than 99.9%.^88^ In an extension of this research, La and Mo of LMO were replaced by Ce or W, and the results suggested that a partial substitution of Ce for La increased the antiviral activity against Φ6.^89^

Pezzotti *et al.* developed a solid-state virucidal bioceramic utilizing sintered Si_3_N_4_, and it exhibited enhanced antiviral capacities against H1N1, HEV71, FCV,^90^ and SARS-CoV-2.^91^ Upon the hydrolysis of Si_3_N_4_ at the surface, reactive nitrogen species (RNS) were generated, which have antiviral properties. RNS can be metabolized by mammalian cells but are toxic to bacteria and viruses. It was observed that polypropylene-grafted methacrylamide (PP-*g*-MAM) possesses excellent antiviral capacities against T7 bacteriophages, upon chlorination.^92^ The composite demonstrated a high melting temperature (161 °C), along with a breaking tensile stress of 7 MPa and breaking strain of 100%. Moreover, its reusability and rechargeability enable its use in protective textiles.

Many other studies have incorporated photoactive compounds, which generate ROS upon light exposure and are capable of high antiviral capacities of more than 5 log reductions against SARS-CoV-2,^70^ T7 bacteriophages,^93–95^ F2 bacteriophages,^96^ feline infectious peritonitis viruses (FPV),^93^ dengue-1 virus (DENV),^97^ and vesicular stomatitis virus (VSV),^97^ and these composites can mainly be used as coatings for personal protective clothing with excellent photostability, reusability, and washability, as well as biocompatibility. Moreover, an antiviral capacity of 2.4–2.8 logarithmic reduction value (LRV) was observed against bovine coronaviruses (BCoV), with the use of peroxotitanium acid/peroxo-modified anatase as the photocatalytic material.^98^ This coating can be applied in cattle breeding environments as well as indoor spaces such as offices and hospital rooms.

**Fig. 7:**
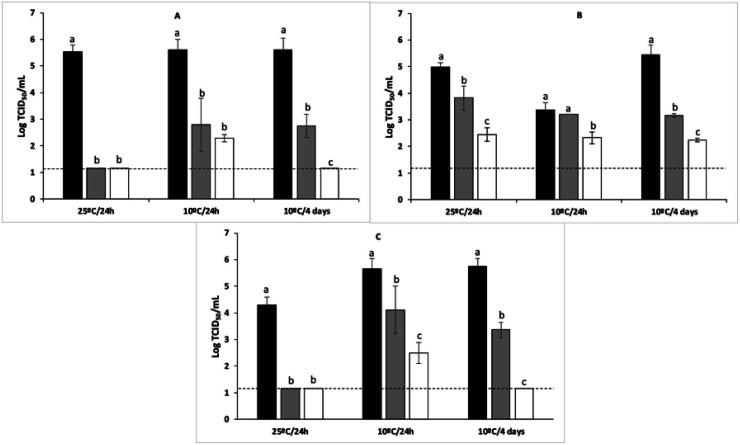
Reduction of murine norovirus titres on blueberries after treating with different coatings ((A) agar coating; (B) alginate coating; (C) agar/alginate coating) at different temperatures and storage times (this figure has been adapted from ref. 101, with permission from Elsevier, copyright 2021). *Black bars: virus control; Grey bars: coating control; White bars: coating Ln. **Dashed lines: detection limit.

Another major application of antiviral coatings is their use in the food industry (Fig. 7). This is of utter importance because consumption of foods contaminated with human enteroviruses, such as hepatitis A (HAV) and human noroviruses (NoVs), can cause severe disease.^99^ Thus, the application of food-grade edible coatings has recently gained attention for controlling the safety of fresh products by functioning as a virucidal barrier and a preservative to reduce spoilage and pathogen attacks. These coatings show enhanced antioxidant capacities (Trolox equivalent antioxidant capacity of 4.5–14.1), and increased tensile strength (11–13 MPa) and elastic modulus (477–1607 MPa).^99–101^

## 4. Overview

The goal of this mini review is to deliver the current state of knowledge, research directions, and applications by examining the most recent developments in antiviral surface coatings and filters and analyzing their efficiencies. Antiviral personal protective equipment, particularly face masks, have become a major industrial focus because aerosols comprising the viruses are smaller, and they are able to pass through most commercial filter masks, increasing the risk of infection. This is where nanotechnology comes in handy, as reusable masks with antimicrobial properties and easy decontamination could be a valuable tool in the fight against virus spread. The antiviral capability of face masks can be improved to reduce the risk of cross-infection or secondary infection during use or handling. Given the current challenges posed by the COVID-19 pandemic, disposable mask recycling could have a significant impact on lowering economic and environmental costs. Recent advancements in this field suggest that nanotechnology has the potential to fundamentally alter the structure and efficacy of current respiratory protection devices.^102^ Moreover, large quantities of masks and PPE must be quickly produced during viral outbreaks, for example, using 3D printing and nano-electrospinning for the fabrication of nanofibers to compose filters.^103,104^

Due to viral adhesion and colonization followed by proliferation with the formation of biofilms,^105^ surfaces in public places such as healthcare centers, long-term care facilities, public transportation, schools, and various businesses are easily contaminated. Traditional disinfection/cleaning methods such as spraying of ethanol (62–71%), sodium hypochlorite (0.1%), or hydrogen peroxide (0.5%) can be used to temporarily remove surface contamination.^106^ However, antiviral surface coatings that repel pathogens *via* non-adhesion or neutralize pathogens with self-sanitizing ability would be the most desirable techniques.^106,107^ Some of the key factors to consider when developing potential coating materials are low toxicity, high efficiency, ease of use, health concerns, and durability.^108^ Nanomaterials such as metal oxides, GOs, and CNTs, as well as bio-nanoparticles such as chitosan, silver, copper, graphene, gold, and silicon nanoparticles, have yielded high antiviral capacities. However, additional research is unquestionably required to develop commercially realized substances.

The current COVID-19 pandemic has already generated a massive amount of new knowledge and rapidly developing technologies. It is now an open question as to what we should expect from the next, more evolved, and potentially far more deadly virus. The goal of materials scientists would be to develop next-generation antiviral agents with unique antiviral characteristics and high antiviral capacities by predicting the next step in viral evolution with the aid of recently published studies. We recognize that it is easier to speculate than to achieve, but the most recent breakthrough provides us with strong confidence that the target viruses and their evolution trajectories are now much more realistic to identify. The search for additional universal antiviral materials, as well as efforts to uncover the universality of common viral receptors, should be pursued with even greater vigor. We hope that this article will aid in the development of more evolved surface coatings and filters to prevent the spread of COVID-19 and other infectious diseases as well as future outbreaks, control epidemics, and avoid highly undesirable pandemic and endemic developments.

The antimicrobial properties of many materials have been extensively studied, but there are far fewer reports on antiviral properties, which is a gap that should be addressed. Moreover, antiviral research requires the sharing of results and data, which is especially important during viral outbreaks. Finally, protective device functional integration and engineering are currently unsystematic, necessitating additional research and study.

## Conflicts of interest

There are no conflicts to declare.

## Supplementary Material
